# Soft Template Electropolymerization of Polypyrrole for Improved pH-Induced Drug Delivery

**DOI:** 10.3390/ijms21218114

**Published:** 2020-10-30

**Authors:** Iryna Liubchak, Matthew T. Lawrence, F. Benjamin Holness, Aaron D. Price

**Affiliations:** Organic Mechatronics & Smart Materials Laboratory, Department of Mechanical and Materials Engineering, Western University, London, ON N6A 5B9, Canada; iliubcha@uwo.ca (I.L.); mlawre28@uwo.ca (M.T.L.); fholness@uwo.ca (F.B.H.)

**Keywords:** electroactive polymers, conjugated polymers, drug delivery, electropolymerization, polypyrrole, inflammation, wound healing

## Abstract

Chronic wounds are characterized by a localized pH change from acidic (healthy) to alkaline (unhealthy), which can be harnessed to act as a switch for drug release from a polymer medium covering the wound for improved healing. To realize this, a new polymer dressing material is needed to help heal chronic wounds. Polypyrrole (PPy) is a biocompatible electroactive polymer that has been proven as a successful drug delivery mechanism, but currently lacks the capacity for scalable clinical applications due to its poor processability. In this study, PPy films with and without microstructures were produced using electrochemical oxidation and subsequently doped with fluorescein, a model drug molecule. To increase the drug loading capacity, microstructures were created through soft template polymerization of pyrrole around hydrogen gas bubbles. Fluorescein release was measured using UV spectroscopy over a pH range of 2 to 11, showing increased release at higher pH values. Microstructured films showed an increased doping capacity compared to flat PPy films, attributed to the increase in drug incorporation sites. The pH-activated release mechanism was shown to be successful and can be applied as a pH-sensitive biosensor and drug delivery system in vitro.

## 1. Introduction

Targeted drug delivery is growing in popularity because it allows an increased localized drug concentration while reducing peripheral side effects. Drug delivery materials must have two functions, clinically relevant carrying capacity and controlled/stimuli-responsive drug release that define when the drug is administered. Furthermore, these methods must be biocompatible and nontoxic. These properties are exhibited by specialized polymers that have been used as carriers in drug delivery systems.

An exciting application of targeted drug delivery is the healing of chronic wounds, which affects up to 6.5 million patients in the United States [1]. Chronic wounds occur when typical interventions fail to close the exposed tissue, this disrupts the natural tissue healing cycle and leaves patients at risk of infection. Chronic wounds commonly occur in patients with comorbidities such as diabetes or obesity [1]. The acidic nature of skin acts as the first line of defence for the body, working to fight against the bombardment of pathogens encountered in everyday life. Many bacteria require a pH value of greater then 6 to proliferate on the skins surface. Chronic wounds have a pH of 7.7±0.3, creating a breeding ground for infection [2]. In well-healing wounds, an acidic environment created by lactic acid due to increased oxygen demands is linked to collagen production and enhances fibroblast proliferation [2]. The switch to an alkaline environment in chronic wounds is explained by the imbalance between tissue regeneration and degradation processes resulted from the impaired blood supply. The degradation of skin tissue is performed via proteolytic enzymes that require an increase in pH values for their activity [3]. Treatment of chronic wounds relies on returning the area to a natural acidic state of healing. This means restoring the wound to the natural acidic skin pH of 6.2–6.6 to prevent infection. Ensuring this defense mechanism is intact is key to maintaining ones health. To mitigate bacterial ingrowth, this study proposes implementing conducting polymers as a wound dressing material for the storage and delivery of drug molecules based off wound pH. These stored drug molecules could be antibiotics to prevent infections or substances that decrease the pH of the wound to minimize reinfection.

Intrinsically conductive polymers (ICP) have overlapping properties between metals and polymers, combining the processability of polymers with the electrical advantages of metals [4]. This is opposed to studies which work to combine polymers and metals into one composition, such as hydrogels and metal nanoparticles [5]. Polypyrrole (PPy) is a biocompatible ICP that has potential applications for controlled drug delivery due to its reduction–oxidation properties [4,6,7,8]. PPy is commonly synthesized using chemical oxidative polymerization through the use of various oxidizing agents [9]. Chemical synthesis results in the formation of PPy particles dispersed in solution, which can be separated and used in their desired application [10]. The major limitation of this method is the formation of a free particle mixture, rather than bulk PPy. An alternative to chemical oxidative polymerization is electrochemical deposition, where the cycling of the potential difference between electrodes placed in a solution of pyrrole monomer and a doping salt causes an oxidative reaction to occur and deposit PPy on the working electrode in the cell. PPy films created using this technique can be easily removed from the electrode and used in various applications [8,11].

It has been shown that switching ICPs between oxidized and reduced states allows the absorption and release of specific molecules [7,8,12,13]. Ion movement in and out of the polymer film is called doping, and dedoping and can be carried out chemically or electrochemically [14]. The doping process is highly dependent on the surface area of the material, a larger surface area equates to an increased number of active sites for drug incorporation. A limitation of electrochemical creation methodologies is the reduced surface compared to PPy particles created chemically, reducing the carrying capacity.

To load a clinically sufficient quantity of drugs into PPy, an increased ion-doping capacity is required. A promising approach to give PPy increased capacity is through the creation of surface micro- and nanostructures [7,15,16,17,18,19,20]. A nanowire network, produced through electrochemical polymerization of pyrrole on a gold electrode, helped to achieve a larger drug loading capacity in the study by Jiang et al. [18]. Studies show improved electrochemical performance from PPy films with microstructures compared to flat films [21]. Uppalapati et al. introduced template-directed and template-free mechanisms for polymerization of conductive polymers with defined microstructures [22]. This novel approach uses amphiphilic molecules as templates for ICP polymerization, called soft template-directed polymerization. This process involves potentiodynamic polymerization of pyrrole around hydrogen bubbles enwrapped by surfactant molecules, as described by Qu et al. [15]. By applying this method and controlling polymerization conditions, defined microstructures of varying shapes and sizes can be created [16,17,19]. Bajpai et al. [17] demonstrated the ability to create such PPy microcontainers to encapsulate fluorescein (FL) molecules. For the following study, a modified soft template-directed method has been employed to create varying surface microstructures on PPy films to investigate the effect in its drug loading and release capability for drug delivery applications with the use of the model molecule, FL.

There are still limitations that prevent broad use of ICPs as drug delivery systems, many of which are related to ion movement through the polymer. The traditional method of ion exchange in ICP experiments is achieved by cycling the potential inside a electrochemical cell [3,8], similar to the creation and doping processes. However, this complex system does not exist in vivo where polymer drug delivery system can be utilized. Therefore, there has been an active search for new stimuli that can trigger the oxidation/reduction reaction in PPy that can act as a substitute for the original electrochemical methods. It has been shown that certain chemical molecules can act as redox reagents and trigger drug release from the conducting polymers [23]. Samanta et al. [10] synthesized PPy nanoparticles that were able to release drug molecules, triggered by a change in local pH. As previously mentioned, the areas around chronic wounds drop from a natural skin pH of 6.5 to 7.7, creating an ideal switching point to act as a feedback mechanism to stimulate drug delivery. Based on the properties of PPy that allow it to respond to pH changes in the local environment, novel drug delivery systems can be developed. This means that the PPy films can be created to deliver medications only when the pH of the wound increases. The protonation/deprotonation reaction that occurs when PPy is in either an acidic or alkaline environment would cause the release of the drug molecule from the polymer matrix. Given the previous research in both PPy drug delivery and film surface modifications there is a need to investigate the relationship between the two. This paper reports a novel fabrication method and characterization of pH-triggered drug release from PPy films with microstructures for increased drug storage capacity for use as a treatment for chronic wounds.

## 2. Materials

(1R)-(-)-10-Camphorsulfonic acid (CSA) (Fisher CAS ^#^35963-20-3), sodium chloride (NaCl, Fisher, CAS ^#^7647-14-5), sodium hydroxide (NaOH, Fisher, CAS ^#^1310-73-2), and TRIS-HCl Buffer pH 7.5 (Fisher, Cat. ^#^15567027) were all use as received. Pyrrole 98% (Sigma Aldrich CAS ^#^109-97-7) was twice distilled before use, 1mol/L hydrochloric acid (HCl, Fisher, CAS ^#^7647-01-0) was diluted to 0.1mol/L, and ethanol 95% (Sigma Aldrich CAS ^#^64-17-5) was diluted to 70%. Fluorescein sodium salt (FL, Sigma Aldrich CAS ^#^518-47-8) was used as received.

## 3. Methods

PPy films were prepared using cyclic voltammetry (CV) in a three-electrode electrochemical cell containing an aqueous solution of 0.5mol/L CSA and 0.2mol/L of Py. All potentials were referenced to a Ag/AgCl reference electrode. A scan rate of 20mV/s was applied for all potential ranges [15,16]. The working electrode (WE) and counter-electrode (CE) were fabricated from stainless steel (SAE 304). The electrochemical response of the films was captured through a custom LabView interface. The area of film polymerization was normalized by covering all sides of the electrode except where polymerization was desired, leaving an exposed electrode area of 10mm×20mm to the polymerization solution. The final polymerization area was measured using calipers to account for any errors and to normalize release data, outlined below in Section 3.5.

### 3.1. Vertical Electrode Configuration

An initial thin PPy base layer was deposited on the WE by cycling the potential between 0–1.2V for 2 cycles. PPy microstructures were formed around hydrogen bubbles appeared at the potential range −0.6–(−1.6)V followed with polymerization at 0–1.2V for either 7 cycles for closed spheres or 2 cycles for open spheres. To create taller structures, 0–1.4V for 3 cycles was applied, followed with 1 cycle of −0.8–(−1.2)V and 4 cycles of 0–1.4V.

### 3.2. Horizontal Electrode Configuration

**Figure 1:**
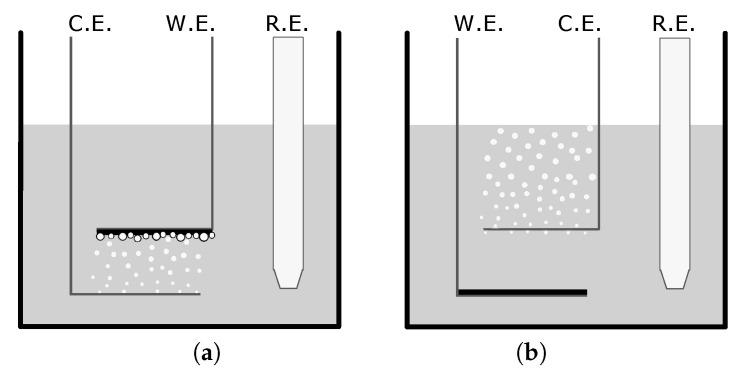
Set-up diagrams depicting the counter electrode (CE), working electrode (WE), and reference electrode (RE) for the creation of (**a**) a microstructured film and (**b**) a flat film in a camphorsulfonic acid/pyrrole solution. The variance in setup allows for the bubble formation and entrapment which creates a variance in surface structure.

The 20mm×70mm electrodes were bent $90^{{}^{\circ}}$ 10mm from the tip of the electrode, providing a horizontal parallel plate configuration, seen in Figure 1. For flat film creation, the CE was placed 5mm above the WE. PPy was deposited on the WE by applying a potential range between 0 and 1.2V for two cycles. To create microstructures, the WE was placed 5mm above the CE. One positive CV cycle between 0 and 1.2V was applied to deposit a flat PPy base layer on the WE. Any hydrogen bubbles that were on either electrode were removed using nitrogen gas. The potential was cycled in a negative range of −0.6 to −1.1V to generate hydrogen bubbles on the WE. After bubble formation, one additional cycle between 0 and 1.2V was applied. After completion, the films were rinsed and stored in deionized water between creation and doping to prevent drying.

### 3.3. Morphology

The morphology of the microstructures was studied using a Keyence VHX-7000 optical microscope and a Hitachi S-3400N V scanning electronic microscope. Before imaging, samples were rinsed with 70% ethanol and subsequently dried in order to preserve the structure of surface features. For feature measurements, the VHX-7000 software was used with a three-point edge detection method selection. The computerized edge detection reduced the human error in the measurements of feature diameter. Four separate locations were examined across the film, totaling $1\;mm^{2}$.

### 3.4. Conductivity

Conductivity was tested on both the flat and microstructured films using a 4-point probe method and a Keithley 2611 Source Measure Unit. The rinsed films were removed from the electrodes and dried overnight prior to conductivity testing.

### 3.5. pH Sensitivity and Drug Loading Capacity

Comparison testing was conducted between flat and microstructured films to determine the FL doping capacity, a model dopant which is easily visualized. Doping was completed using CV between 0 and 2.4V for 6 cycles at 20mV/s, in an aqueous 0.1mol/L NaCl, and 0.001mol/L FL solution. Doped PPy films were washed with deionized water and immersed in buffer solutions with pH of 2.0,7.5,8.0 and 11.0 at room temperature. After two hours of release the films were removed and the solutions were examined via UV–Vis spectroscopy. The absorbance peaks of FL were recorded for each sample. Flat and microstructured films were tested in triplicate at each pH value to compare both pH release and doping capacity. The Beer–Lambert law was used to calculate the concentration from the release solutions, from this data the amount of FL can be calculated in $mg/m^{2}\;$by dividing by electrode polymerization area.

## 4. Results and Discussion

### 4.1. Vertical Electrode Configuration

The first experiment used a vertical electrode set-up, described by Qu et al. [15]. Pyrrole was polymerized around hydrogen bubbles attached to the PPy base layer on the WE. The negative potential was terminated when sufficient bubbles were covering the surface of the PPy film, found to be −1.1V, upon which time polymerization in the positive potential range was continued. The switching between positive and negative potentials could be repeated multiple times to create stacked bubble tower-like features. This study shows that structures can grow directly on PPy films. The fabrication of microstructures on previously deposited PPy layers is novel as opposed to the creation of microstructures directly on the stainless steel.

**Figure 2:**
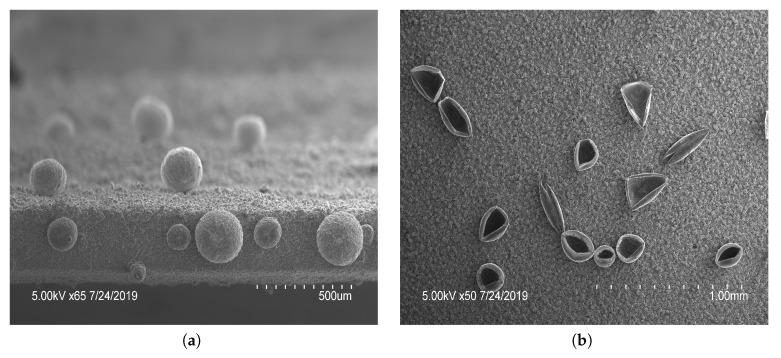
Scanning electron microscopy images of polypyrrole (PPy) films with features: PPy microstructures formed by potential cycling 2 cycles of 0–1.2V, followed by 2 cycles of −1.6–(−0.6)V and (**a**) 7 or (**b**) 2 cycles between 0and1.2V. The number of cycles in the last stage of polymerization dictated the open or closed nature.

**Figure 3:**
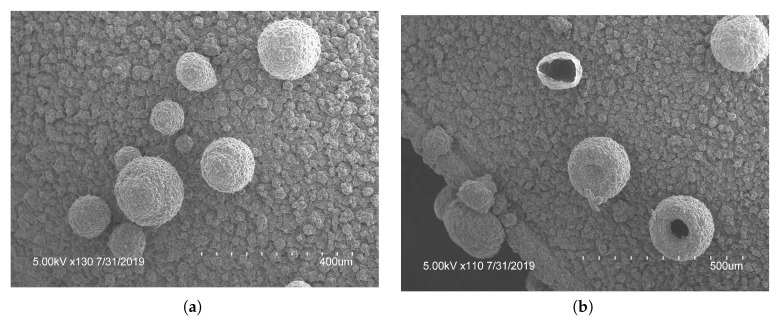
Scanning electron microscopy images of different polypyrrole (PPy) microstructures; (**a**) closed and (**b**) open structures were produced by applying 3 cycles of 0–1.4V, 1 cycle of −0.8–(−1.2)V to create bubbles, followed by 4 cycles of PPy polymerization 0–1.4V. Showing the variance of structures capable on a single film.

The microstructures created on vertical electrodes included both open and closed spheres, seen in Figure 2 and Figure 3. As shown in Figure 2, the increased number of cycles allowed a longer polymerization time and had an increased number of closed spheres. A total of 7 cycles was used to fully close the sphere and a minimum of 2 cycles was needed to create open features of sufficient thickness to withstand rinsing. An intermediate number of cycles between 2 and 7 yielded increasingly closed structures. It was shown that applying a larger potential range (0–1.4V) during electropolymerization resulted in varying types of microstructures to form (Figure 3). Qu et al. recorded a taller structure being created with the increased potential voltage [16]. However, in our work a larger potential range was found to not drastically increase the height of the structures created using these parameters, but resulted in an increased variability in the structures created, seen in Figure 3b, having both open and closed structures on a single film. As a result, the potential range of 0 to 1.2V was used in subsequent tests for consistency. The closing of microstructures has potential application in drug delivery devices due to the increased storage volume, which warrants further testing in future studies. These results show that there is a large degree of variability in the creation of microstructures. To reduce the variability and increase the density of such microstructures, a horizontal electrode method was adopted using similar CV parameters tested previously.

### 4.2. Horizontal Electrode Configuration

**Figure 4:**
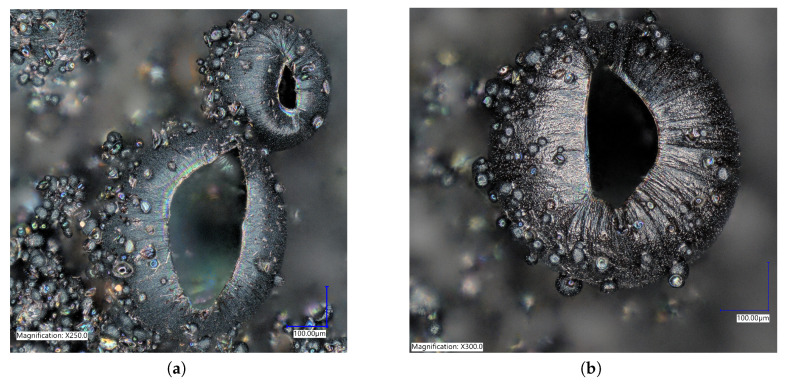
Optical microscopy pictures at (**a**) ×250 and (**b**) ×300 of polypyrrole films created using bent electrodes. These shows the stacked and hierarchical structures that increased the ion doping sites for increased doping capacity.

With electrodes arranged in a vertical configuration, it was observed that hydrogen bubbles would detach from the film on the WE when the hydrostatic force was larger than the electrostatic force. Meaning larger bubbles were difficult to create accurately or repeatedly. To reduce bubble detachment, the electrodes were bent at $90^{{}^{\circ}}$. This created a horizontal parallel plate configuration of electrodes parallel with the surface of the solution during polymerization, trapping any bubbles formed. With this configuration, the electrodes were 5mm apart as compared to the vertical electrodes being 10mm apart. As the electrodes were closer together in the horizontal electrode set-up, the number of cycles required was decreased. To improve consistency, a voltage range of 0 to 1.2V was used for the remaining tests. Polymerization occurred more rapidly with horizontal electrodes. Due to improved bubble attachment, the horizontal electrodes caused coalescence if the negative potential was larger than −1.1V or the applied scan rate was faster than 20mV/s. When the hydrogen bubbles coalesced into one mass, the porous structures were connected and grew exponentially, subsequently decreasing the surface area and lowering the drug loading potential. To mitigate this, the voltage range and scan rate were kept at the previously mentioned limits. With the horizontal electrode configuration there was a large increase in the density of the microstructures created, seen in Figure 4a. The horizontal electrode configuration also allowed for the creation of hierarchical microstructures, seen in Figure 4. The addition of smaller microstructures allowed an increased surface area for doping. PPy spheres as small as 6.4μm were observed throughout the film. The smaller bubbles were most likely from the last cycle of polymerization when the lower CE was hydrolyzing water which created microbubbles that were released and moved up towards the WE. These smaller bubbles attached to preexisting polymerized structures on the WE, created in the previous cycle. Future studies are required to fully explore the relationship between the CV parameters and the formation of bubbles using horizontal electrodes. The novel horizontal plate set-up yielded a unique microfeatured surface structure and an increased surface area, ideal for drug delivery.

### 4.3. Morphology

**Figure 5:**
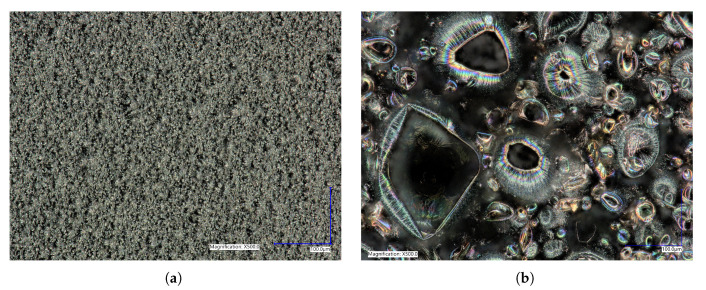
Surface morphology of (**a**) flat polypyrrole (PPy) films compared to (**b**) microstructured PPy films at 500X magnification showing the effectiveness of the horizontal plate method for the creation of round microstructures. Image (**b**) is an example of what was used for the feature size counting outlined in Figure 6.

**Figure 6:**
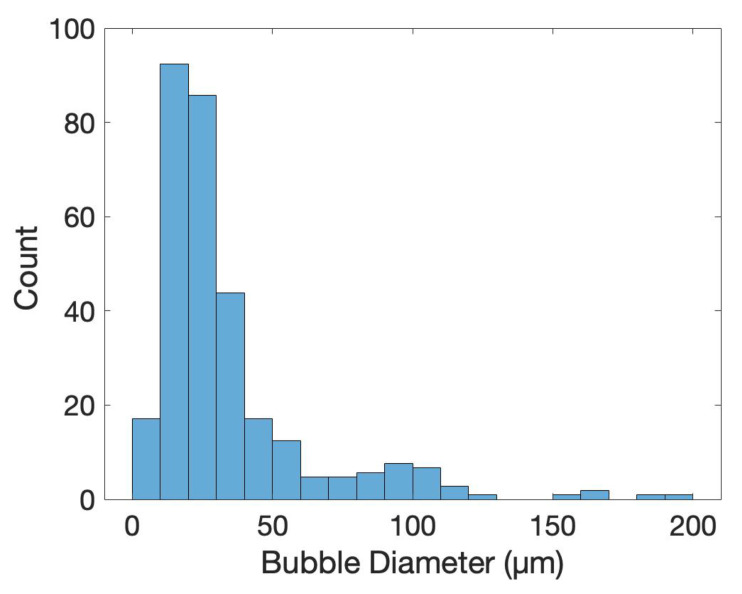
A histogram of bubble size collected in four separate locations on a microstructured polypyrrole film, totalling $1\;mm^{2}$. The creation methodology of bubble entrapment lead to a large amount of bubbles with diameters between 10 to 30mm, seen in Figure 4 and Figure 5.

To characterize the difference between flat and microstructured films, the surface of each was imaged using identical microscope settings, seen in Figure 5. The microstructured films showed a large number of bubbles of various sizes, the histogram in Figure 6 shows the distribution of bubble diameter on the surface of the film for $1\;mm^{2}$. A total of 322 bubbles were measured with an average diameter of 34μm. The larger bubbles were between 50 to 200μm with smaller bubbles between 10 to 50μm attached to the surface of the larger bubbles. The coalescence of bubbles on the WE was difficult to control and resulted in a large variance in bubble size. Nevertheless, the films presented above showed a increased density and smaller diameter compared to previous creation methods, showing the success of the novel horizontal plate setup.

### 4.4. Conductivity

The difference in preparation methods leads to a drastic difference in the surface structure of the films, which can impact the conductivity. The 4-point probe conductivity measurements showed average sheet resistances of 408.56 ± 40.21 Ω/sq for flat films and 252.30 ± 49.72 Ω/sq for microstructured films. The improved electrical impedance performance of the microstructured films can be attributed to the increase in exposed surface area provided by the features [21].

### 4.5. pH Sensitivity

**Figure 7:**
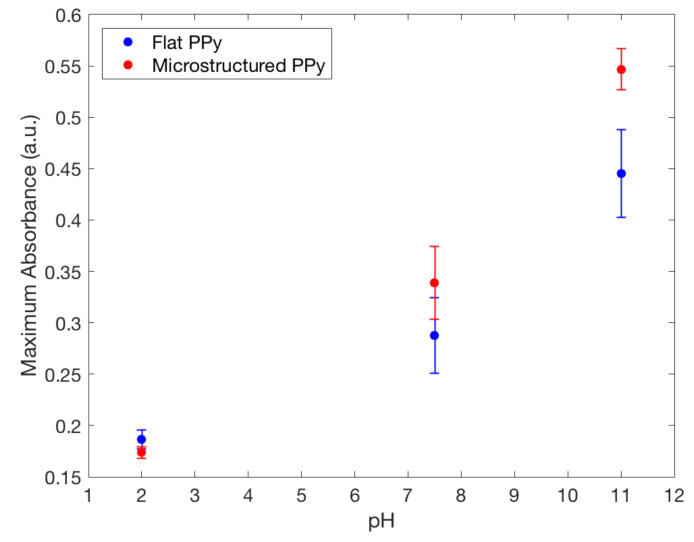
Maximum fluorescence spectra of fluorescein released from flat and microstructured PPy film in: TrisHCl buffer solution with pH 7.5, 0.1mol NaOH solution with pH 11.0 and 0.1mol HCl solution with pH 2.0, showing the increased doping capacity of microstructured films compared to flat.

The loading of a model drug FL into the film is based on the ion-doping process, similar to the electropolymerization of Py. For initiating the doping, a positive potential was applied to the electrochemical cell, resulting in the oxidation of the PPy film allowing a positively charged polymer to bind with a negatively charged FL from the FL/NaCl electrolyte solution. It is important to note that cationic drug incorporation into polymer matrix mainly relies on the interactions with a primary anionic dopant [20], not studied in this paper. The results from the doping and pH release tests show that FL was released in both the 7.5 and 11 pH solutions at room temperature after two hours (Figure 7). At pH 2.0, both flat and microstructured films had similarly low release values, whereas at pH 11 the largest amount of FL was released. These results show a clear switching method for drug release using pH and follow the results previously described by Samanta et al. with FL favouring larger release at higher pH values [10]. For use in chronic wounds, the drop in pH from physiological skin of 6.2–6.6 to 7.4–8.0, seen in chronic wounds, would act as a stimulus for drug release from PPy [1]. The mechanism of drug release is based on the protonation/deprotonation reaction of the PPy backbone in low and high pH, respectively [10]. Deprotonation of the polymer decreases the positive charge and drug anions, such as FL, can be released. On the other hand, protonation increases the overall positive charge of PPy causing FL to become entrapped on the polymer backbone during the oxidation reduction that is created while doping. Incorporation of positively charged compounds is based on the hydrophobic and electrostatic interactions with primary anion dopant [3].

### 4.6. Drug-Loading Capacity

**Figure 8:**
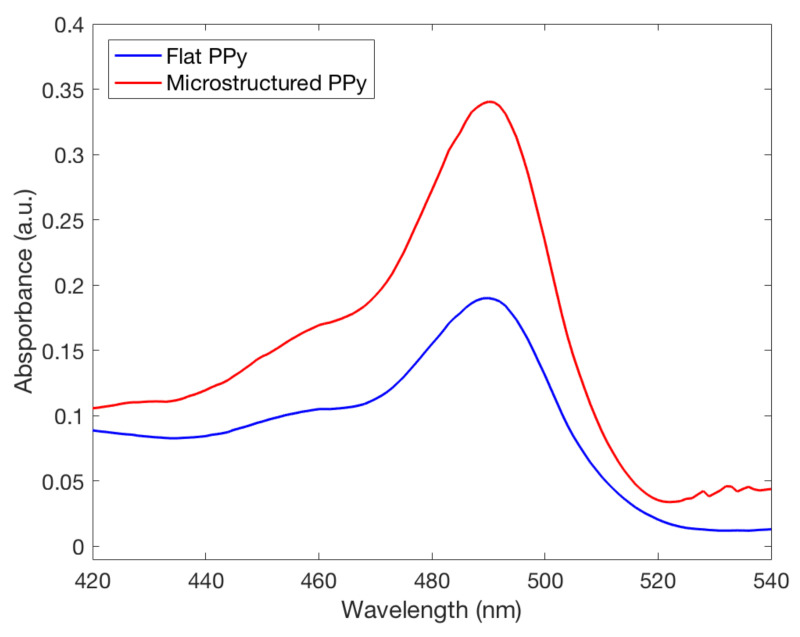
Fluorescence spectra of fluorescein released from both flat and microstructured polypyrrole films in a solution with a pH of 8.0, commonly seen in chronic wounds. The microstructured film can be seen having an increased doping capacity at this pH.

FL was released from both types of films in the neutral and basic pH mediums they were placed in. A higher absorbance peak of FL was recorded by UV spectrometry in a solution where microstructured PPy films were stored, seen in Figure 7. This finding indicates that a larger amount of FL was incorporated and released by the PPy films with microstructures. Using the Beer–Lambert equation it was seen at pH 11 the microstructured films had released $0.354\pm 0.010\;mg/m^{2}$ compared to $0.273\pm 0.033\;mg/m^{2}$ for flat films, showing that there was a significant difference between the two structure types for drug delivery at pH 11. To confirm clinical use, the pH value of 8 was used as a severe case wound pH. The average absorbance was plotted against wavelength, seen in Figure 8. A doubling of maximum absorbance can be seen between the microstructured and flat films, taking this data and using the Beer–Lambert equation and normalizing it by area it was found that there was $0.144\pm 0.053\;mg/m^{2}$ release from microstructured films compared to $0.107\pm 0.019\;mg/m^{2}$ for flat films. Samanta et al. described only a 13% and 15% FL release from PPy nanoparticles in pH 7.4 and 8, respectively, showing that there could be an increased amount of FL stored in our films unable to be released due to the reduced pH. This also correlates to the higher FL release at pH 11 data presented earlier, a pH not studied by Samanta et al., showing the need for a larger pH for increased % release from PPy [10]. Although the pH 8 flat and microstructure release values are within one standard deviation, there was a 34% average increase of release at pH 8, showing that there was a definitive increase in release despite a large variance in data. This is low compared to the results found by Luo et al. who described a 10X increase in FL release from nanoporous PPy films but this was using electrical stimulation, not seen naturally inside the human body, and therefore not directly applicable with this passive system [7]. The multistep doping and release process used in this study has not been optimized for such small changes in concentrations and future studies will work to reduce the variability, allowing the detection of smaller changes at lower pH values. The outlined results show that hierarchical microstructures allowed increased loading and release capabilities at high pH values. The increased capacity means that the highly porous designs have the potential to deliver medications at a clinically relevant dosages depending on the severity of the wound.

The microstructured films had an increase in surface area which corresponded to increased doping sites for storage of the FL molecules. The oxidation reaction during CV corresponds to a loss of electrons from the polymer and demonstrates that the incorporation of drug anions (ion doping) in PPy is feasible, whereas the reduction reaction represents dedoping in basic solutions. Given the increased absorption values of the microstructured films, it can be said that the introduction of such structures improves the ion-doping capacity of PPy films by providing additional sites for ion incorporation. Thus, the improved electrochemical properties inherent to PPy films having these microstructured surfaces makes them a more suitable storage material for incorporated drug molecules for chronic wound treatment.

Future testing will include varying model drugs of various size and charge to better elucidate this increased release. Antiseptic treatments such as olyhexanide and antibiotics such as quinolones, microlides, and aminoglycosides used to treat infection have an increased activity in alkaline environment possessed by chronic wounds and are exciting drug options for future studies [2,24,25]. Pairing this type of coating with a hydropolymer coating or scaffold would combine two drug delivery methods for increasingly controlled and sustained release device, responsive to pH. Previous research has shown the capabilities of hydrogels in wound healing [5,26]. This data shows preliminary improvements in microstructure films using FL as one such model drug. In this study, the film mass difference was too small to effectively normalize the difference between the flat compared to the microstructured films. The ability of PPy to release loaded molecules while being switched between oxidized and reduced states offers the proposal of novel activation methods, other than pH. There is potential for various triggers for drug delivery which can be tested. Release studies at 37 ${}^{{}^{\circ}}$C would be a valuable future endeavor as it has been noted that there is a small increase in conductivity with an increase in temperature [27]. Future efforts will focus on increased control of feature size as well as the stacking of hierarchical features.

## 5. Conclusions

In this study, the improvement of ion-doping capacity was shown in PPy films with microstructures prepared by soft-template directed electropolymerization. The novel horizontal plate method created microbubbles with diameters between 6.4 to 192μm which lead to an increase in surface area and active sites for drug incorporation. Enhanced electrochemical performance and electrical conductivity due to the presence of hierarchical microstructures allows these films to be used for the development of novel sensors to treat chronic wounds. The fluctuating pH surrounding PPy activated drug release from both types of films. These results indicate that PPy films are viable candidates for controlled drug delivery systems. Specifically, it was shown that negatively charged FL can be loaded into PPy films and released in basic pH solutions. Testing at the clinically relevant pH value of pH 8 showed an increased drug capacity in the microstructured films compared to flat, that can be further optimized for specific medications. It was concluded that microstructure-modified PPy films are more suitable for use in drug delivery applications because they demonstrated increased drug doping and release capability of FL. Improved drug doping capacity in polymer delivery systems allows creators to load a clinically relevant dosage of medication to provide a sufficient concentration at the site of placement. This study demonstrates a novel horizontal plate creation method that allowed the creation of complex microstructured films. It was shown that PPy can be used as a drug-doped dressing material for the use in chronic wounds for pH activated release.

## Abbreviations

The following abbreviations are used in this manuscript.

CE	Counter-Electrode
CV	Cyclic Voltammetry
FL	Fluorescein
NaCl	Sodium Chloride
ICP	Intrinsically conductive polymers
PPy	Polypyrrole
RE	Reference Electrode
SEM	Scanning Electron Microscope
WE	Working Electrode
